# A novel technique for large-fragment knock-in animal production without ex vivo handling of zygotes

**DOI:** 10.1038/s41598-023-29468-1

**Published:** 2023-02-08

**Authors:** Manabu Abe, Ena Nakatsukasa, Rie Natsume, Shun Hamada, Kenji Sakimura, Ayako M. Watabe, Toshihisa Ohtsuka

**Affiliations:** 1 Department of Animal Model Development, Brain Research Institute, Niigata University, 1-757 Asahimachidori, Chuo-Ku, Niigata, 951-8585 Japan; 2 Department of Biochemistry, Faculty of Medicine, University of Yamanashi, Yamanashi, 409-3898 Japan; 3 Institute of Clinical Medicine and Research, Research Center for Medical Sciences, The Jikei University School of Medicine, Chiba, Japan

**Keywords:** Biological techniques, Biotechnology, Genetics

## Abstract

CRISPR/Cas-based genome editing has dramatically improved genetic modification technology. In situ electroporation called genome editing via oviductal nucleic acid delivery (GONAD), which eliminates the need for ex vivo embryo handling, is technically the simplest method for gene transfer and can be performed in laboratories without developmental engineering expertise including micromanipulation techniques. However, the use of this method remains challenging in the case of large-fragment knock-in, such as gene expression cassettes. Adeno-associated viruses (AAV) act as donor DNA for homologous recombination in infected cells, including rodent embryos. In this study, we demonstrated simultaneous electroporation of AAV donors and CRISPR/Cas9 components into embryos to create knock-in animals, and successfully generated knock-in rats carrying a gene cassette with a length of 3.0 kb using a small number of animals and in situ electroporation. These findings indicate that this technique is an efficient high-throughput strategy for producing genetically modified rodents and may be applicable to other animal species.

## Introduction

Recent advances in genome editing technology have made the genetic modification, including knockout and knock-in mutations, a relatively simple technique in various animal species^1–7^. Generally, specialized equipment and sophisticated developmental engineering techniques are required to produce genetically modified animals. However, the development of electroporation-based methods simplified the process significantly^8–13^. Although these methods are beneficial, producing genetically modified animals containing large DNA fragments (multiple kilobase sequences), such as gene expression cassettes, remains challenging since developmental engineering that requires in vitro manipulation of embryos is often a difficult technique. For instance, the developmental characteristics of rats differ from those of mice, and collecting embryos for ex vivo gene manipulation in some rat strains is difficult^14,15^. Improved genome editing via oviductal nucleic acid delivery (*i*-GONAD) is an in situ electroporation method developed to overcome these challenges and appears to be the simplest technique^16–21^. *i*-GONAD has reported successful knockout, point mutation knock-ins in rats, and reporter knock-ins of less than 1 kb in mice, but multikilobase size knock-ins have not been reported in either mouse or rat *i*-GONAD.

On the contrary, AAV is known to function as a donor DNA for homologous recombination (HR)^22,23^. After ex vivo AAV infection of embryos, a method for knock-in of multikilobase sequences was developed^24–27^. Due to their biological nature, AAV1 and AAV6 can pass through the zona pellucida and infect mouse and rat embryos, acting as donors by entering the nucleus of the embryos^24,25,28^. However, electrical pulses delivered to embryos via electroporation have been shown to induce structural changes in the plasma membrane and the zona pellucida and to make microholes^8^. Therefore, the current study considered the possibility that AAV along with CRISPR/Cas9 components could be immediately and physically introduced into cells by electric pulses and function as donor DNAs. Furthermore, we tested whether electroporation could achieve kilobase-sized knock-ins without prior AAV infection. The technique developed in this study was aimed to facilitate multikilobase insertions and, when combined with conditional knockout and inducible expression systems (such as the Cre/loxP recombination system and the doxycycline-inducible system), it was expected to advance the understanding of the molecular mechanisms underlying higher-order life phenomena.

## Results

### Site-specific knock-in by ex vivo electroporation with AAV vectors

**Figure 1:**
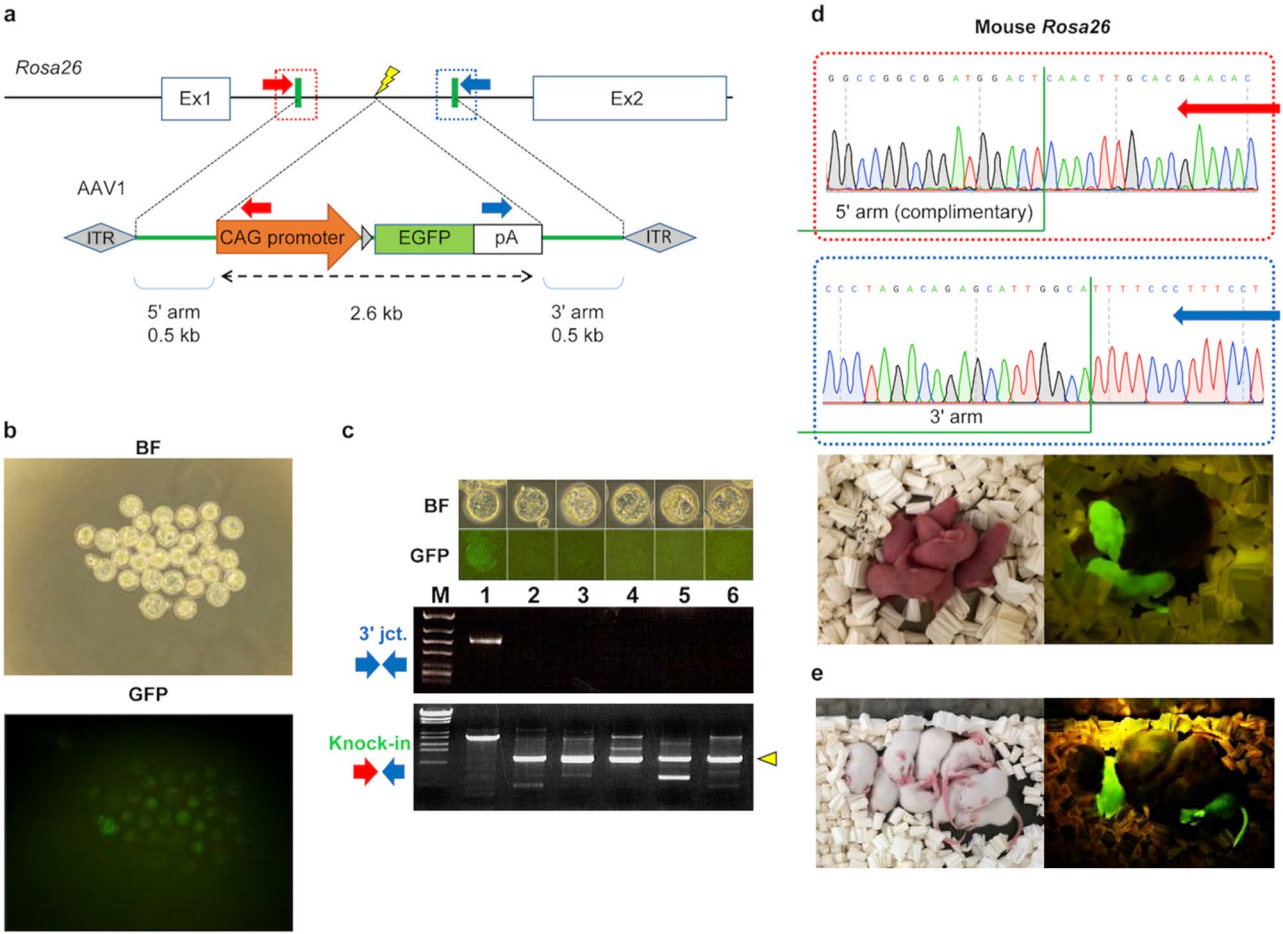
Generation of knock-in mice by simultaneous electroporation of Cas9 RNP and AAV vectors ex vivo. (**a**) Targeting strategy for CAG-EGFP knock-in into the mouse *Rosa26* locus. Red and blue arrows indicate PCR primers for 5′ and 3′ junction genotyping. Green vertical bars represent 5′ and 3′ junctions. A small gray triangle shows a loxP sequence. ITR: inverted terminal repeat; pA: rabbit beta-globin polyadenylation signal. (**b**) Representative fluorescent microscopy of embryos electroporated with CRISPR RNP and AAV1-CAG-EGFP vector. BF: bright-field microscopy. **(c**) PCR products amplified from genomic DNAs of individual blastocysts. Insertion of CAG-EGFP cassette in the *Rosa26* locus was detected from an embryo (No.1) expressing EGFP fluorescence. 3′ expected band size: 0.6 kb; knock-in expected band size: 3.6 kb; wild-type expected band size (yellow triangle): 1.1 kb. Sanger sequencing confirmed that correctly edited 3′ junction of the *Rosa26* locus, although the 5′ junction was incorrect in this embryo (data not shown). Faint PCR products were detected in several samples; however, they are thought to be due to nonspecific amplification because they are not detected as signals by Sanger sequencing. M, λ-*Sty*I (lower) or ϕX174-*Hinc*II (upper) digested DNA marker (Supplementary Fig. S4). (**d**) Correct HR in embryos was confirmed by Sanger sequencing. ICR pups expressing EGFP fluorescence were obtained after ex vivo electroporation of CRISPR RNP and AAV1 donors. (**e**) The germline transmission of the edited *Rosa26* locus was confirmed in the next generations.

The present investigation analyzed the potential of AAV vectors to serve as donor DNA for HR without prior infection. First, unlike what has been previously reported, CRISPR RNPs were simultaneously electroporated with AAV donors containing a fluorescent protein gene expression cassette targeting the *Rosa26* locus using serotype 1 (Fig. 1a), which is known to serve as donor DNAs. A few embryos expressing fluorescent proteins were found when AAV vectors were washed out and developed in vitro to blastocysts. Genomic PCR of the embryo showed that HR was directed to the *Rosa26* locus (Fig. 1b,c) although it is not ruled out the possibility of transgene integration at other desired sites via either random integration or via transgene integration at off-target DNA double-strand break. Additionally, transplantation of the embryos into pseudopregnant mice produced knock-in mice that expressed fluorescent proteins throughout the body, and correct HR in knock-in embryos and mice was confirmed by Sanger sequencing (Fig. 1d). Mating these individuals confirmed germline transmission (Fig. 1e).

Using AAV vectors of serotypes 2, 5, and 6, CRISPR RNPs were also electroporated into embryos, and the knock-in efficiency was confirmed using genomic PCR. PCR products from knock-in embryos were substantially detected in all serotypes, although we could not prove exact homologous recombination by bilateral homology arms in a single blastocyst. (Supplementary Fig. 1). It is well known that different AAV serotypes have varying degrees of infectivity for mouse zygotes^24,25,28^. Correspondingly, we found that serotypes 2 and 5 are less effective for infecting zygotes (Supplementary Fig. 2a). In addition, electroporation of AAV vectors in the absence of CRISPR RNPs did not result in physiological infection of any serotypes (Supplementary Fig. 2b). These findings suggest that AAV vectors function as donor DNAs in ex vivo electroporation and are introduced into the cells via a mechanism distinct from that of physiological infection.

### Generation of knock-in mice by in situ electroporation

**Figure 2:**
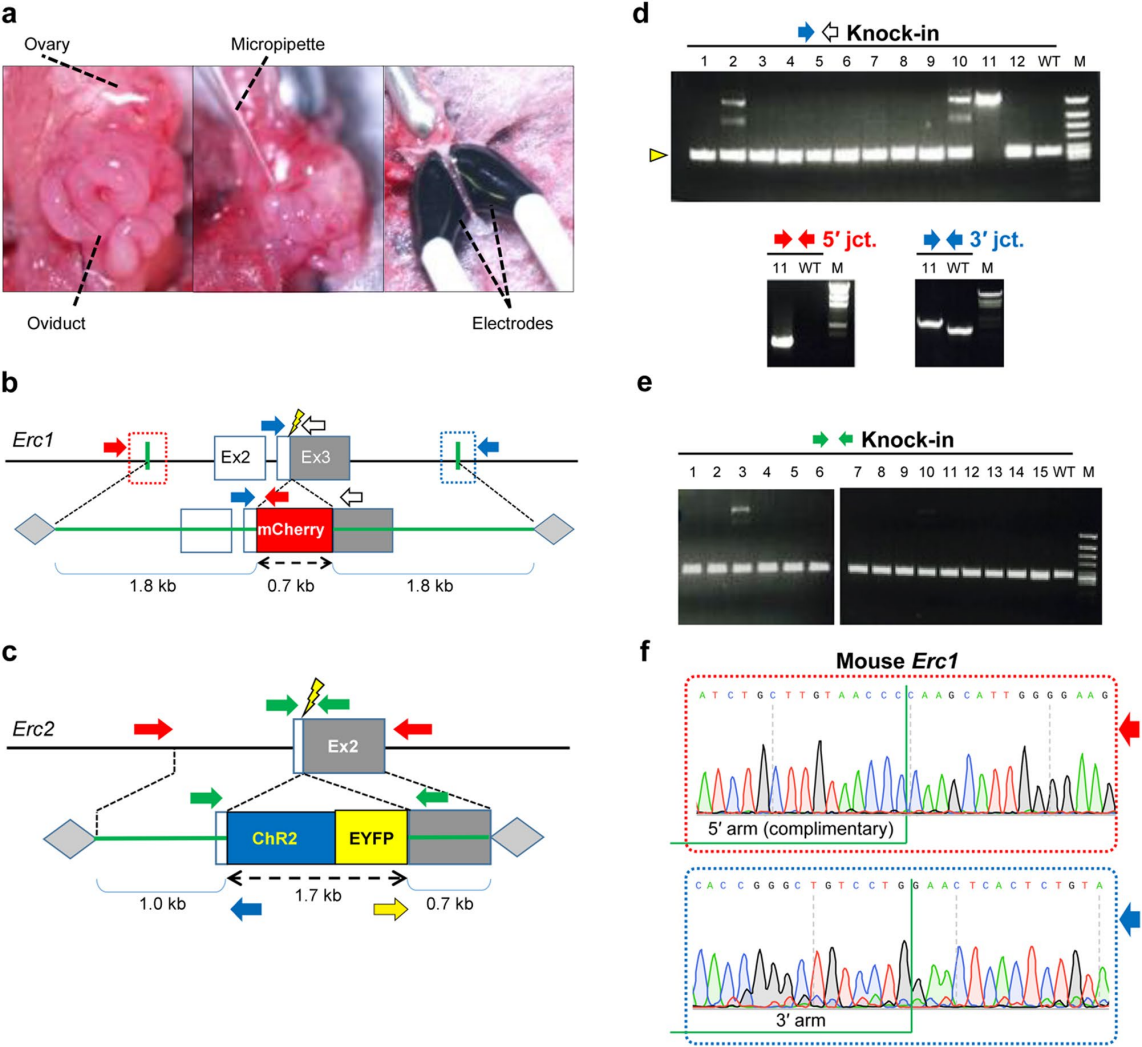
Generation of knock-in mice by in situ electroporation using AAV1 donors. (**a**) Procedure of in situ electroporation. Our protocol is based on the *i*-GONAD method, which has been well described in other reports^17, 44^. Left: exposed ovary and oviduct are shown; middle, CRISPR components are instilled into the oviduct lumen using a glass micropipette; right, the entire oviduct covered with a small wet paper is electroporated using tweezer-type electrodes. (**b**, **c**) Schematic of strategy to insert a mCherry fluorescent reporter or ChR2-EYFP cassette into endogenous *Erc1* (ELKS) or *Erc2* (CAST) locus, respectively. Arrows indicate the PCR primers. (**d**, **e**) Confirmation of insertions by genomic PCR. Two *Erc1* knock-in heterozygous (No. 2 and No. 10) and one homozygous (No.11) (in **d**), and one *Erc2* knock-in heterozygous (No. 3) (in **e**) mice were obtained. Wild-type expected band size (yellow triangle): 0.3 kb; knock-in expected band size: 1.1 kb; 5′ expected band size: 2.1 kb; 3′ expected band size: 2.3 kb in **d**. Knock-in expected band size: 2.1 kb; wild-type expected band size (yellow triangle): 0.4 kb in **e**. M, ϕX174-*Hinc*II digested (in **d**, upper and in **e**) or λ-*Sty*I digested (in **d**, lower) DNA marker; WT, wild-type mice. The cropped gel images were grouped from different parts of the same experimental result in e. (Supplementary Fig. S5,S6) (**f**) Representative sequencing chromatograms for correctly edited mCherry-ELKS knock-in mice.

We anticipated that knock-ins would also be possible by in situ electroporation in the oviduct, i.e., *i*-GONAD^17^ (Fig. 2a), since we had discovered that AAV donors work in embryos without pretreatment to infect them with the virus. In the above experiment, the use of serotypes 2, 5, and 6 did not prove accurate homologous recombination by bilateral homology arms, so we decided to use serotype 1 for the following experiments. The AAV1-CAG-EGFP vector was initially used to create knock-in mice using outbred ICR strains. However, for some reason, we could not produce pups expressing fluorescent proteins. It is well known that recombination efficiency varies from locus to locus. We attempted knock-in of functional molecular sequences at the other two target loci, *Erc1* and *Erc2,* to verify whether the same method can be used to generate knock-in mice in the widely used C57BL/6N (B6N) inbred mouse strain^29–31^. Ex vivo electroporation preliminary experiments revealed that higher AAV concentrations typically result in higher knock-in efficiencies (data not shown). We, therefore, carried out electroporation for each target locus using the highest AAV solution concentration (2.1–3.2 × 10^11^ viral genome copy (vg)/mL). The AAV1 vector harboring 0.7 kb mCherry sequence flanked by two 1.8 kb homology arms (4.3 kb in total) for *Erc1*, and the vector harboring 1.7 kb ChR2-EYFP cassette sequence flanked by 1.0 kb and 0.7 kb homology arms (3.4 kb in total) for *Erc2* were introduced by in situ electroporation (Fig. 2b,c). As a result, it was possible to obtain mice with precise HR at the two loci (Fig. 2d,e), and both germline transmissions were verified. Correct HR in mCherry-ELKS knock-in mice was confirmed by Sanger sequencing (Fig. 2f).

Additionally, it was established that in the brains of knock-in mice, mCherry and the *Erc1* gene product ELKS form a functional fusion protein (Supplementary Fig. 3a,b) and are detected in the stratum lucidum of the hippocampal CA3 region and the molecular layer of the cerebellum as expected (Supplementary Fig. 3c). These findings suggest that, although the efficiency tends to be lower than in earlier studies that were successful in large-fragment knock-in by ex vivo electroporation, it is still feasible to produce knock-in mice with AAV donors harboring functional gene cassettes by in situ electroporation^24,25^. Table 1 provides a summary of the knock-in efficiency. Unexpectedly, all knock-in animals obtained by in situ electroporation were produced by exact homologous recombination, and no animals were born in which incorrect recombination was detected.

**Table 1:** Knock-in efficiency by in situ electroporation.

Species (strain)	*Locus*	Pregnant female	Pups obtained	Knock-in pups (%)
Mouse (ICR)	*Rosa26*	2	27	0 (0.0)
Mouse (B6N)	*Erc1*	2	14	3* (21.4)
Mouse (B6N)	*Erc2*	2	28	2 (7.1)
Rat (LH)	*Rosa26*	5	49	3 (6.1)
Rat (SD)	*Rosa26*	3	10	1 (10.0)
Rat (LE)	*Thy1*	2	19	1 (5.3)

*One of these pups harbored a homozygous mutation.

### Generation of knock-in rats by in situ electroporation

**Figure 3:**
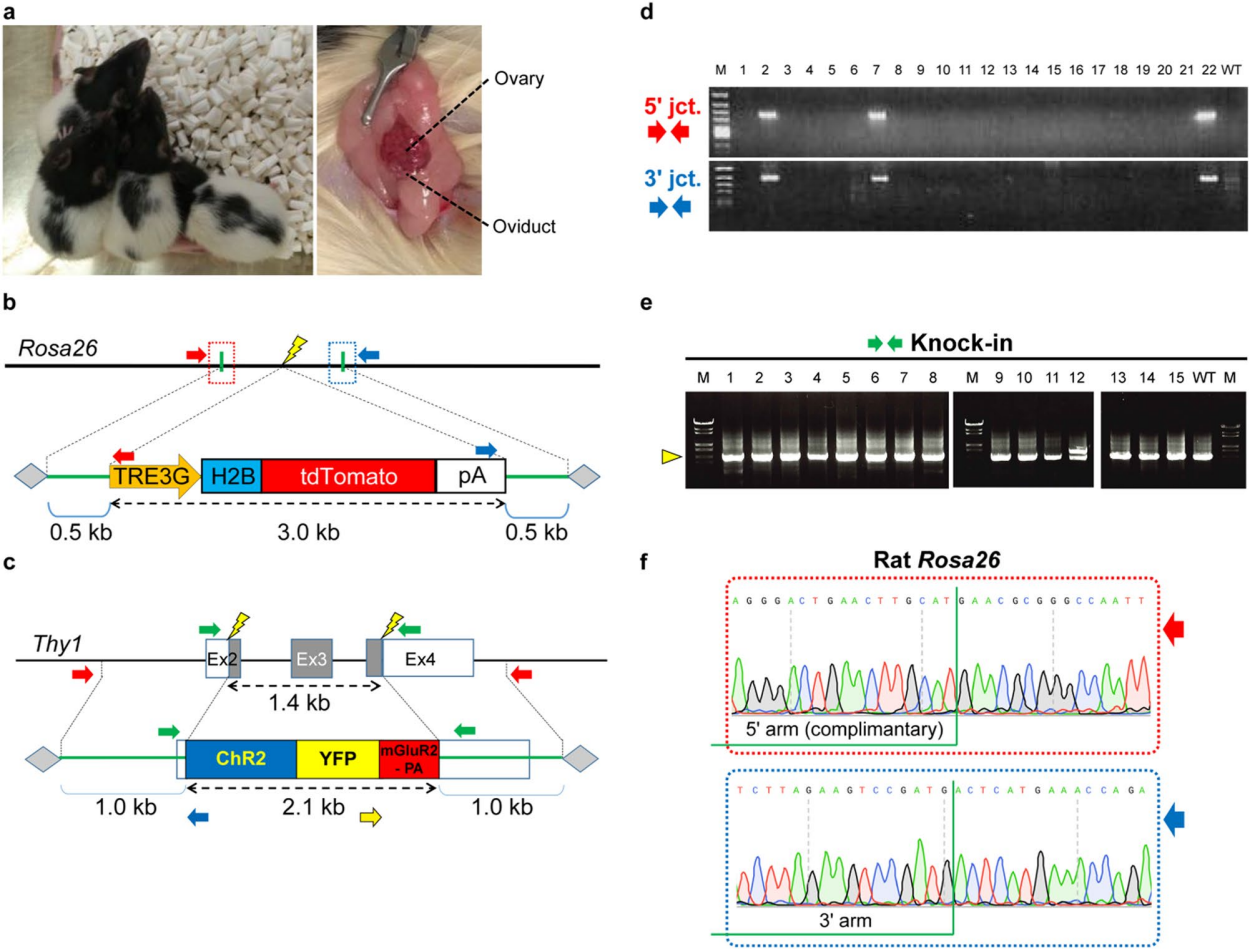
Generation of knock-in rats carrying a gene cassette by in situ electroporation of AAV1 donors. (**a**) Left, Establishment of a breeding colony of LH rats for in situ electroporation; right, the exposed ovary and oviduct of the rat. (**b**, **c**) Schematic of strategy to insert a tetO-H2B-tdTomato or a ChR2-YFP-mGluR2-PA cassette into endogenous rat *Rosa26* or *Thy1* locus, respectively. TRE3G, the third generation of tetracycline operator (tetO) sequence; H2B, histone H2B. Arrows indicate the PCR primers. (**d**, **e**) Confirmation of insertion using PCR. Three *Rosa26* knock-in heterozygous LH rats (No. 2, No. 7, and No. 22) (in **d**), and one *Thy1* knock-in heterozygous LE rat (No. 12) (in **e**) were obtained. 5′ expected band size: 0.7 kb; 3′ expected band size: 0.8 kb in (**d**). Knock-in expected band size: 2.5 kb; wild-type expected band size: 1.8 kb (yellow triangle) in (**e**). M, ϕX174-*Hinc*II digested (in **d**) or λ-*Sty*I digested (in **e**) DNA marker; WT, wild-type rats. (Supplementary Fig. S7,S8). The cropped gel images were grouped from different parts of the same image in e. (**f**) Representative sequencing chromatograms for correctly edited tetO-H2B-tdTomato knock-in rats.

The *i*-GONAD method has been shown to be effective for introducing in/del mutations and point mutations in rats^18,32^. As a result, we attempted to generate knock-in rats through in situ electroporation with AAV donors. We had set up a breeding colony in the outbred Lister Hooded (LH) strain to produce gene-modified rats (Fig. 3a). AAV1 vector with a 3.0 kb tetO-H2B tdTomato cassette flanked by two 0.5 kb homology arms targeting the rat *Rosa26* locus (Fig. 3b) was injected with CRISPR/RNP into oviducts of LH rats and electroporated. As a result, we were able to find neonates in which HR was precisely occurring. Under the same conditions, a closed colony Sprague–Dawley (SD) rat strain was electroporated. Consequently, it led to the generation of rats with HR (Fig. 3b,d) and germline transmission. In addition, using inbred Long–Evans (LE) rats, we successfully inserted a 2.1 kb ChR2-YFP-mGluR2-PA cassette^33^ into the endogenous *Thy1* gene (Fig. 3c,e). Although the cause is unknown, an unexpected single nucleotide mutation (c.-48G > A) was found in the 5′ UTR sequence of the edited *Thy1* gene. Correct HR in tetO-H2B tdTomato cassette knock-in rats was confirmed by Sanger sequencing (Fig. 3f). These findings show that it is possible to generate knock-in rats with multikilobase cassette sequences without ex vivo handling of zygotes.

## Discussion

This study demonstrates that knock-in rodents with multikilobase sequences can be generated using a very simple method. Many previous studies relied on sophisticated equipment, such as pronuclear injection, to produce genetically modified animals^7,34–37^. The *i*-GONAD method is an in situ electroporation technique that avoids handling embryos ex vivo and has been developed as a more practical method to get around these technical restrictions. It successfully inserted a 783-bp long T2A-mCitrine cassette using lssODN^17^, but it has not been reported to be successful in knocking in a large fragment beyond that^21^. In the current study, we successfully introduced AAV vectors along with CRISPR/Cas9 components through in situ electroporation, resulting in a knock-in of roughly 3 kb. According to reports, ssAAVs can package multiple kilobases up to 4.9 kb^38^. Previous research using ex vivo electroporation demonstrated that AAV donors with a total length of 0.2 kb used as HA were successfully knocked-in^24^, implying that our method could knock-in more than 4 kb. Fragments larger than 3 kb may provide valuable opportunities for in vivo functional analysis of genes of interest since they can contain gene expression cassettes consisting of promoters for gene expression control, open reading frames, and polyadenylation sequences. In addition, sequential HR may exceed knock-in fragment length restrictions based on the AAV vector packing capacity^22^. On the other hand, in this study we have not tested for the possible existence of transgenes that are not targeted to the desired locus. As with other knock-in strategies using genome editing technologies, such unexpected mutations may need to be verified, for example, by using Next Generation Sequencing, or other methods.

Consequently, this strategy will offer fantastic opportunities to clarify the molecular mechanisms of higher-order life phenomena. The current study found that the simultaneous electroporation of CRISPR/Cas9 components and AAV donor vectors into zygotes inside oviducts (i.e., the procedure without pretreatment to infect embryos with AAV) was as valid as in the previous study. Although the molecular mechanism by which AAV functions as donor DNAs in HDRs was not made explicit in this study, it is very likely that AAV moved into the cytoplasm and even into the nucleus in a way distinct from the typical infection pathway. However, as in previous studies, we cannot rule out the possibility that AAV vectors served as donor DNAs through infection due to transient exposure to electroporation solutions containing high concentrations of AAV^24–26^. On the other hand, the results presented in this study suggest that even if that mechanism exists in some proportion, it is not the primary pathway to act as donor DNA in the method applied in this study. The first point is that the CRISPR/Cas9 components used in this study were Cas9 proteins forming a complex with guide RNA. This component is expected to act on genomic DNA immediately after electroporation introduction to cause DSBs. Homologous recombination events would not be anticipated to occur in the absence of donor DNAs if AAV vectors only infect the zygote after a delay. AAV serotypes AAV2 and AAV5, which were previously believed to be noninfectious to zygotes, were electroporated ex vivo and produced knock-in blastocysts at a frequency comparable to that of infectious AAV1 and AAV6. Thus, the outcomes of this experiment show that AAV vectors had already colocalized in the nucleus and served as donor DNAs by the time CRISPR/Cas9 components caused DSBs. Applying a poring pulse to introduce CRISPR/Cas9 components into the zygotes would create microholes in the zona pellucida and oolemma under the experimental conditions we used. AAV, a relatively small (about 22 nm in diameter) viral species, may flow into the cytoplasm and nucleus almost simultaneously with CRISPR/Cas9 components because microholes of 20–120 nm in diameter may form instantly after electroporation at the plasma membrane^39,40^.

In the current study, in situ electroporation allowed inserting large gene fragments into rats and mice. Even with the advancement of genome editing technology, knocking in large fragments in rats is more challenging than in mice. Success with ex vivo electroporation has only recently been reported. Rats have long been used as a model for human pathology and as an excellent experimental animal for pharmacological research^24,26^. Rats are about 10 times larger than mice and require a large housing environment and costs. Therefore, the number of animals used can be significantly reduced by omitting the process of collecting embryos. In situ electroporation, compatible with the 3R principle, enables the knock-in of large fragments, which is a significant advance.

It is known that the infectivity of AAV to embryos varies among animal species^24^. This method does not require ex vivo embryo manipulation. Knock-in is possible irrespective of infectivity, which suggests that it is likely to be more applicable to a broader range of animal species than previously reported techniques. Most importantly, it does not require the sacrifice of experimental animals. The procedure is anticipated to be helpful for genetically modifying experimental animals with strict ethical requirements, such as the common marmoset, which is a small New World primate.

The current study used in situ electroporation of AAV donors and CRISPR/Cas9 components into embryos to create knock-in animals with large-fragment insertions. Both large-scale projects and small-scale lab experiments can produce functional knock-in animals that contain gene expression cassettes with the help of this methodology. Investigating experimental variables like the concentration of AAV donors or the make-up of CRISPR solutions could lead to further improvements in knock-in efficiency.

## Methods

### Animals

B6N mice, ICR (CD1) mice, SD rats, and LE rats were obtained from The Jackson Laboratory Japan (Yokohama, Japan). LH rats (No. 0873) were obtained from the National Bio Resource Project for the Rat (NBRP-Rat, Kyoto, Japan). ELKS conditional KO mouse was made floxed ELKS mouse crossed with E3N-Cre mouse^41,42^. Mice and rats were kept in a standardized laboratory animal facility (lights on 8:00 a.m. to 8:00 p.m.; room temperature and humidity in the ranges of 22 °C ± 2 °C and 50% ± 20%, respectively) with free access to water and a standard laboratory diet (Oriental NMF, Oriental Yeast, Tokyo, Japan). The Animal Experiment Committee of Niigata University and University of Yamanashi approved all animal experiments used in this study animals (approved numbers, SA01126 and A30-21, respectively), and they were all carried out in accordance with ARRIVE guidelines for the care and use of laboratory also all methods were carried out in accordance with relevant guidelines and regulations. For rat anesthesia, a mixture of three anesthetics was intraperitoneally injected at the rate of 1.275 ml/kg body weight; 2.0 ml midazolam (5 mg/ml; Midazolam, Sandoz, Tokyo, Japan), 1.875 ml medetomidine hydrochloride (1 mg/ml; Domitor, Nippon Zenyaku Kogyo, Fukushima, Japan), and 2.5 ml butorphanol tartrate (5 mg/ml; Betorphanol, Meiji Seika Pharma, Tokyo, Japan) were mixed. Consequently, rats were injected with midazolam, medetomidine hydrochloride, and butorphanol tartrate at rates of 2.0 mg/kg, 0.375 mg/kg, and 2.5 mg/kg, respectively. For mouse anesthesia, the mixture was fivefold diluted with saline and approximately twice the rate of anesthetic was injected intraperitoneally compared to rats. After operation, atipamezole hydrochloride (5 mg/ml; Antisedan, Nihon Zenyaku Kogyo), diluted tenfold with saline, was administered at the rate of 0.75 ml/kg or 5.0 ml/kg body weight intraperitoneally for rat or mice, respectively, and awakening was performed on a 37 °C heating plate.

### Production of AAV vectors

AAV vectors were prepared with the AAVpro Helper Free System (AAV1, #6673, Takara Bio, Shiga, Japan), AAVpro Packaging Plasmid (AAV2, #6234; AAV5, #6664; AA6, #6665, Takara Bio), and AAVpro 293T Cell Line (#632273, Takara Bio). All AAV vector plasmids were constructed by cloning PCR fragments corresponding to the target locus and knock-in sequence into the pAAV-CMV vector between EcoRV and BglII restriction sites, removing CMV promoter, b-globin intron, and hGH polyA. The arms of homology of the mouse *Rosa26*, *Erc1*, and *Erc2* genes were amplified by PCR from genomic DNA of B6N strain. The arms of homology of the *Rosa26* or *Thy1* genes in rats were amplified from the genomic DNA of LH or SD, respectively. PCR fragments of the amplified arms of homology and cloned knock-in sequences and the pAAV vector were seamlessly integrated into a single vector by In-Fusion cloning technology (#639648, Takara Bio). All plasmids were cloned and prepared in large quantities using *E. coli* DH5α (#9057, Takara Bio). Following the manufacturer’s instructions, 293T cells were transfected with an AAV plasmid and packaging plasmids using Xfect Transfection Reagent (#631318, Clontech). AAVs were extracted and concentrated by AAVpro Purification Kit (All Serotypes) (#6666, Takara Bio). Viral genome copies were estimated using the AAVpro Titration Kit (#6233, Takara Bio) and the Thermal Cycler Dice Real Time System III (TP950, Takara Bio).

### Ex vivo manipulation of mouse embryos

Superovulated female mice were obtained by induction with an intraperitoneal injection of 150 IU/kg PMSG (ASKA Pharmaceutica, Tokyo, Japan) and an injection of 75 IU/kg hCG (ASKA Pharmaceutical) after 48 h. In vitro fertilization using fresh sperm from the male mice was conducted according to previously described procedures with minor modifications^43^. Before electroporation, zygotes in the pronuclear stage were gathered and kept in Human Tubal Fluid Medium (HTF, BioSafety Research Center, Kobe, Japan). Embryos in the 2-cell stage after the transduction of AAV donors and CRISPR components were transferred into the oviducts of pseudopregnant ICR mice.

### Preparation of CRISPR solutions

The gRNA duplexes were prepared by annealing synthetic crRNA and tracrRNA in the ratio of 1:1, which were commercially obtained from IDT (Coralville, IA, USA). All target sequences for crRNA applied in this study are shown in Supplementary Table 1. The gRNA duplexes and Cas9 protein were incubated (Alt-R S.p. Cas9 Nuclease V3, IDT) for 5 min at room temperature to assemble the CRISPR/Cas9 RNP. To prepare CRISPR solution for ex vivo electroporation, CRISPR/Cas9 RNP were mixed with AAV in Opti-MEM I Reduced Serum Media (Thermo Fisher Scientific, MA, USA). The final concentrations of components were 0.3 µg/mL Cas9 protein, 30 µM gRNA duplexes, and 3.0 × 10^10^ vg/mL AAV donors. CRISPR/Cas9 RNPs were diluted with Opti-MEM and mixed with AAV solution for in situ electroporation. The final concentrations of components were 1 µg/mL Cas9 protein, 30 µM gRNA duplexes, and 2.1–3.2 × 10^11^ vg/mL AAV donors.

### Ex vivo electroporation

The TAKE method^10^ was applied to deliver CRISPR components to zygotes of mice ex vivo using an electroporator NEPA21 (NEPA GENE, Chiba, Japan). Briefly, pronuclear-stage embryos were transferred into the 5 mm gap-electrode (CUY520P5, NEPA GENE) filled with 50 µL CRISPR reagent containing Cas9 protein, gRNA, and AAV donor. The poring and transfer pulse were set as previously described by Kaneko et al.^10^. After electroporation, zygotes were cultured to be embryos in the 2-cell stage.

### In situ electroporation

The *i*-GONAD method was used to deliver CRISPR components to mouse and rat zygotes in situ. The exposed oviducts of an anesthetized female animal (0.7 days postcoitum) were injected with 1–1.5 µL CRISPR reagent using a glass micropipette. Following injection, electroporation was performed using NEPA21 and tweezer-type electrodes (#CUY652-3, NEPAGENE). The poring and transfer pulse were set as previously described in other reports^44^. The animals were observed recovering from anesthesia and being returned to their home cages following electroporation.

### Genotyping analyses

The automated DNA extraction system was used to extract genomic DNA from tail biopsies or whole blastocysts (GENE PREP STAR PI-80X, KURABO, Osaka, Japan). The PCR products amplified with specific primer sets were directly sequenced. Primers for genotyping analysis are shown in the Supplementary Table 2. For genotyping of mouse* Rosa26* and rat *Thy1* loci, nested PCR was performed using the external primers as follows: 5′-GCTCTCGGGGCCCAGAAAAC-3′ and 5′-GACTTCTAAGATCAGGAAAG-3′ (mouse *Rosa26*); 5′-ATGACATTCGCTGTCATAAC-3′ and 5′-CAGCAGAGAGAACACATATC-3′ (rat *Thy1*).

### Immunoblotting and immunohistochemistry

Anti-ELKS monocolonal antibody was made by Intégrale Co., Ltd. In detail, mice were immunized mouse ELKS 115-142 a.a. synthesized peptides which was low similarity regions for ELKS family protein, CAST, and hybridoma clones were established. Hybridoma selection was performed using Enzyme-Linked Immuno-Sorbent Assay (ELISA) with each antigen peptides. To reject cross reactive antibodies, a second ELISA assay was performed against the CAST peptide. Subsequently, the ELKS-specific hybridomas were purified using protein G columns (GE Healthcare). The cerebellar homogenates (20 µg) or brain sections (thickness, 50 µm) from mCherry-ELKS knock-in mice were analyzed by western blotting or immunofluorescence microscopy as described previously^45^. We used the following primary antibodies: anti-ELKS and anti-RFP for immunoblotting and anti-RFP and anti-NeuN for immunohistochemistry.

## Supplementary Information

Supplementary Information.

## Data Availability

The datasets generated and analyzed during the current study are available in the DNA Data Bank of Japan repository. Accession numbers and persistent links are listed as follows. LC732341; https://getentry.ddbj.nig.ac.jp/getentry?database=na&accession_number=LC732341. LC732342; https://getentry.ddbj.nig.ac.jp/getentry?database=na&accession_number=LC732342. LC732343; https://getentry.ddbj.nig.ac.jp/getentry?database=na&accession_number=LC732343. LC732344; https://getentry.ddbj.nig.ac.jp/getentry?database=na&accession_number=LC732344. LC732345; https://getentry.ddbj.nig.ac.jp/getentry?database=na&accession_number=LC732345. LC732346; https://getentry.ddbj.nig.ac.jp/getentry?database=na&accession_number=LC732346.
