# Supplementary material for: A novel technique for large-fragment knock-in animal production without ex vivo handling of zygotes
Source: Sci Rep. 2023 Feb 8;13:2245. doi: 10.1038/s41598-023-29468-1 (PMC9908863; doi:10.1038/s41598-023-29468-1)
Supplement: Supplementary file 1 — Supplementary Information. [file 41598_2023_29468_MOESM1_ESM.docx]

**Supplementary information**

**A novel technique for large-fragment knock-in animal production without *ex vivo* handling of zygotes**

Manabu Abe^1*,#^, Ena Nakatsukasa^1, #†^, Rie Natsume^1, #^, Shun Hamada^2^, Kenji Sakimura^1^, Ayako M. Watabe^3^, and Toshihisa Ohtsuka^2^

^1^Department of Animal Model Development, Brain Research Institute, Niigata University, Niigata 951-8585, Japan

^2^Department of Biochemistry, Faculty of Medicine, University of Yamanashi, Yamanashi 409-3898, Japan

^3^Institute of Clinical Medicine and Research, Research Center for Medical Sciences, The Jikei University School of Medicine, Chiba, Japan

#These authors contributed equally to this work.

†Deceased

***Corresponding author**

Manabu Abe

Department of Animal Model Development

Brain Research Institute

Niigata University

1-757 Asahimachidori

Chuo-ku Niigata 951-8585, Japan

Phone: +81-25-227-0621

Fax: +81-25-227-0816

E-mail: [manabu@bri.niigata-u.ac.jp](mailto:manabu@bri.niigata-u.ac.jp)

**Supplementary figures**

**
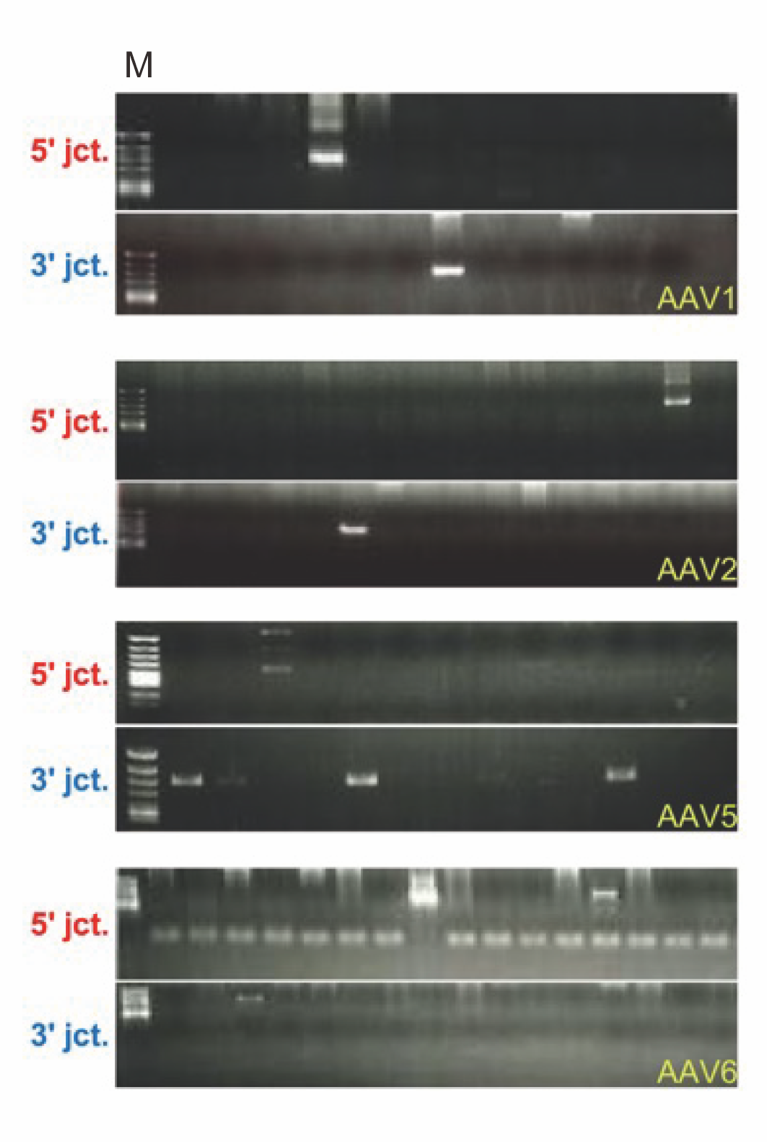
Figure S1**

**Supplementary Fig. S1.** **Genomic PCR of embryos transduced with each serotype of AAV vectors**

PCR products from knock-in embryos were substantially detected in all serotypes, including serotype 2 and serotype 5, which have low infectivity described below. M, φX174-*Hinc*II digested. 5′ expected band size: 0.6 kb, 3′ expected band size: 0.6 kb.

**Figure S2**

**
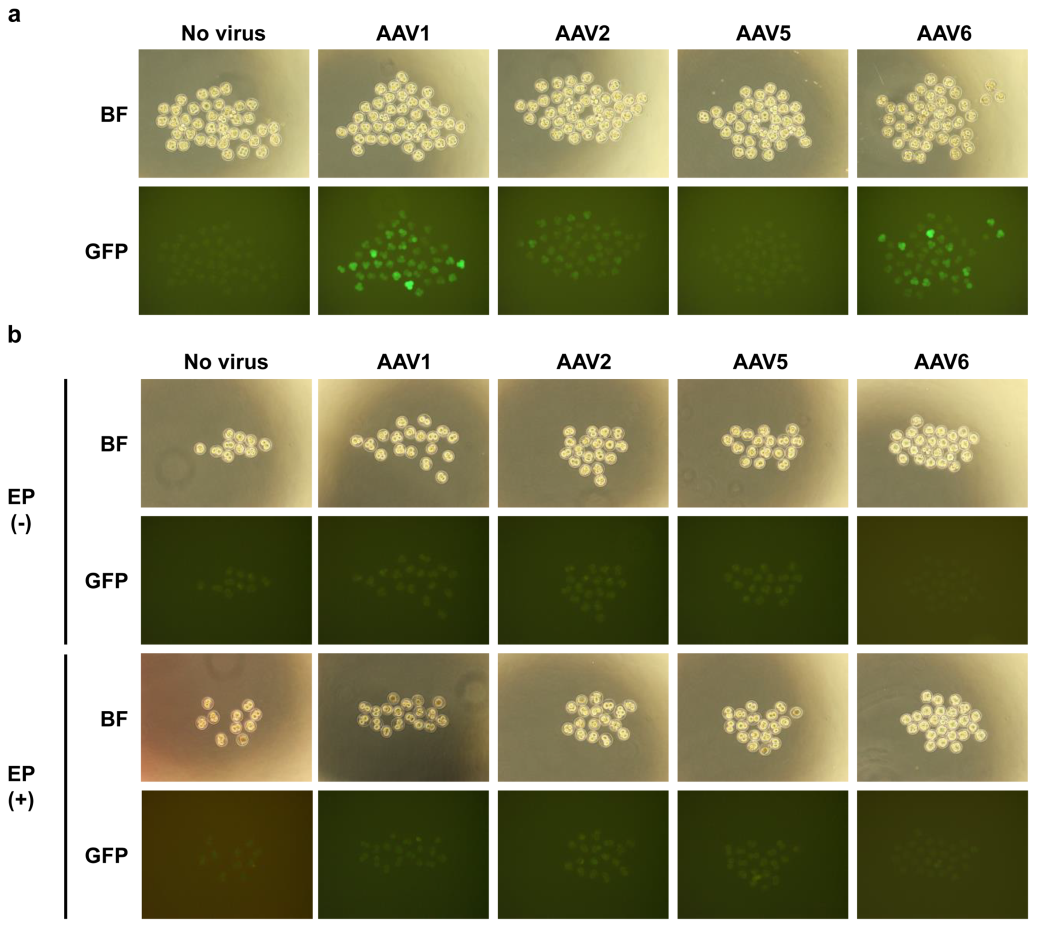
**

**Supplementary Fig. S2. The ability of AAV serotypes to infect mouse embryos and failure of physiologic infection by electroporation of AAV**

**a.** We verified infectivity by exposing embryos to higher concentrations (3.0 × 10^10^ vg/mL) of fluorescent protein-expressing AAV for 48–72 hours, although AAV concentrations of approximately 0.1–1.0 × 10^8^ vg/mL were tested in previous reports. The results showed that serotype 1 and serotype 6 were infectious, as previously reported, but that serotype 2 and serotype 5 were less infectious even at high concentrations.

**b.** AAV serotype 1 and serotype 6, which should be infectious by exposure to embryos, did not express fluorescent protein when the virus solution was washed off immediately after electroporation, indicating that physiological infection had not occurred under this condition. Likewise, serotype 2 and serotype 5 were not infectious either.

**Figure S3**

**
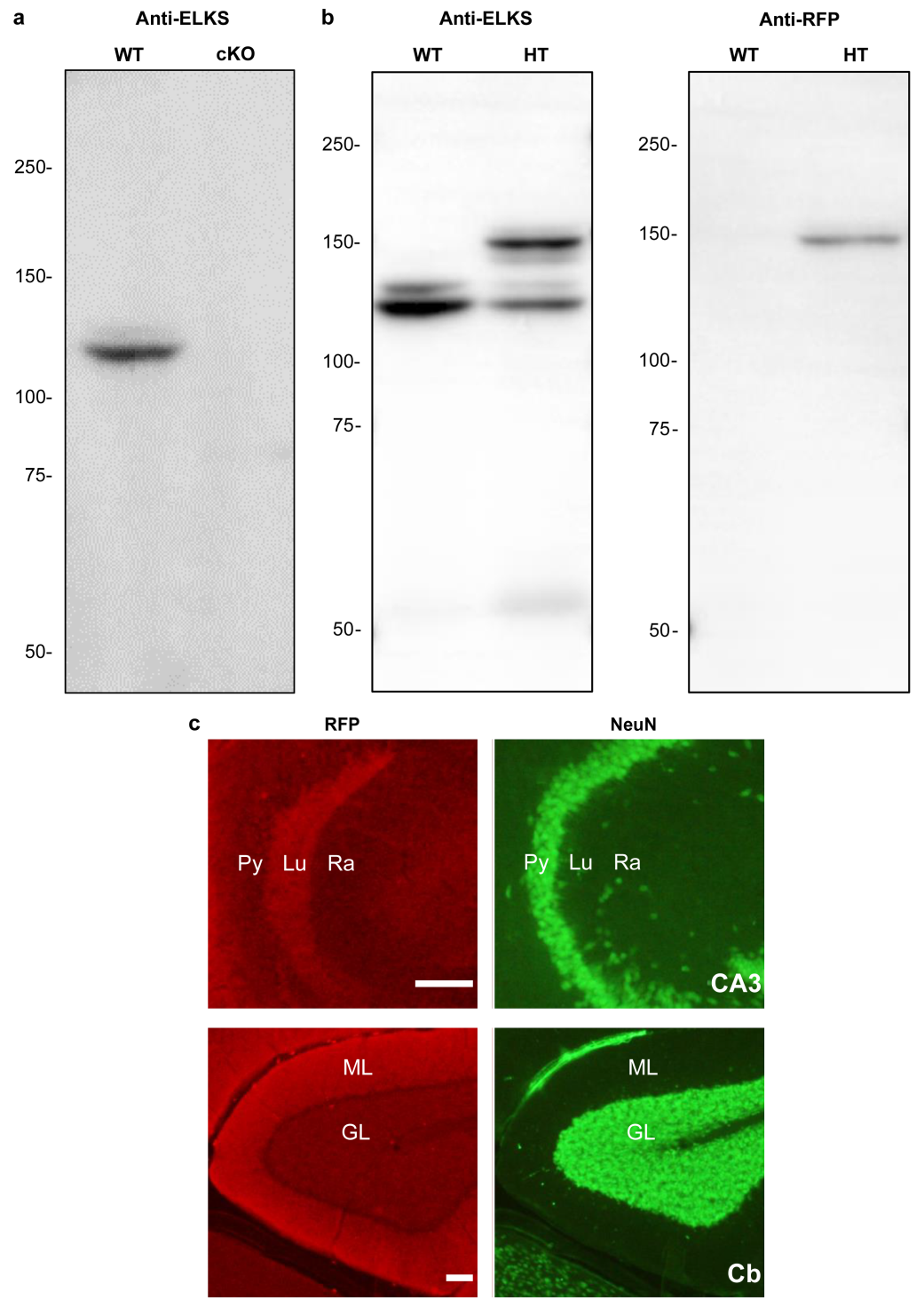
**

**Supplementary Fig. S3.** **mCherry-ELKS knock-in mice**

**a.** Validation of novel anti-ELKS monoclonal antibody. The specificity of anti-ELKS monoclonal antibodies was confirmed by western blot using ELKS conditional KO (cKO) mouse brains. Western blot of WT and cKO cerebellar homogenates was performed with the ELKS monoclonal antibody. The band intensity at about 120 kDa disappeared in the cKO.

**b.** The cerebellar homogenates from wild type (WT) and mCherry-ELKS hetero knock-in mouse (HT) were analyzed by western blot using the indicated antibodies. Both anti-ELKS and anti-RFP antibodies detected mCherry-ELKS fusion protein. The fusion protein expression appears comparable to that of endogenous ELKS protein.

**c.** Immunohistochemistry using the RFP and NeuN antibodies revealed the expression of the fusion protein in the stratum lucidum (Lu) of the hippocampal CA3 region and the molecular layer (ML) of the cerebellum (Cb), as previously described. GL, granular layer; Py, pyramidal cell layer; Ra, stratum radiatum; Scale bars, 100 μm.

**Supplementary tables**

| **Supplementary Table S1. crRNA target sequences for each gene** | | | | | |
| --- | --- | --- | --- | --- | --- |
| Species | *locus* | | Sequence (5′ to 3′) | | MIT score |
| Mouse | *Rosa26* | | CGCCCATCTTCTAGAAAGAC | | 75 |
| Mouse  Mouse  Rat  Rat  Rat | *Erc1*  *Erc2*  *Rosa26*  *Thy1* (left)  *Thy1* (right) | | CACTTCCATACATGGTTGCA  ATCACAGAAGAAAAATGTAC  GAGTCTTTCTGGAAGATAGG  GGAGAGTGATGCTGATGACT  TCACAGAGAAATGAAGTCCG | | 76  47  64  65  60 |
|  | |  | |  | |

| **Supplementary Table S2. Sequences of primers for genotyping** | | | |
| --- | --- | --- | --- |
| Species | *Gene* | Primer | Sequence (5′ to 3′) |
| Mouse  Mouse  Mouse  Rat  Rat | *Rosa26*  (5′ junction)  *Rosa26*  (3′ junction)  *Erc1*  (Knock-in)  *Erc1*  (5′ junction)  *Erc1*  (3′ junction)  *Erc2*  (Knock-in)  *Rosa26*  (5′ junction)  *Rosa26*  (3′ junction)  *Thy1*  (Knock-in) | Forward  Reverse  Sequencing  Forward  Reverse  Sequencing  Forward  Reverse  Forward  Reverse  Sequencing  Forward  Reverse  Sequencing  Forward  Reverse  Forward  Reverse  Sequencing  Forward  Reverse  Sequencing  Forward  Reverse | CTTGCCATTGGCTCGTGTTC  TCCATATATGGGCTATGAAC  AGAAAGGTATTGCAACACTC  CCTCTTCTCTTATGGAGATC  GACTTCTAAGATCAGGAAAG  TAGTCATCTGGGGTTTTATG  GACTCTTCCCCAGATAAAGG  TTGTTCGCCGATGACCTAAG  CGACACATCAGCTTGGTTTG  TTCACGTAGGCCTTGGAGCCGTAC  AAACCTCTCCTTGATATCAG  GACTCTTCCCCAGATAAAGG  GAAGCAGAAGCGTTCCAGAC  CCTTTAATCCTAGCACTCAG  AAGATGCAATGCAATCATAG  CATTAAGGGACTGGATATTC  GCTCTCGGGGCTCAGAAAAC  CCAGCTTACTTACCATGTCAGATC  TGTAACCATTATTCATTCAGGCGC  AGAAAAGCCTTGACTTGAGG  GCTACAGCCTCGATTTGTGG  CCTCTTCTCTTATGGAGATC  TGTAGACCAGGCTGGGCTAG  GGGCCCAACCAGTCACAGAG |

**GEL BLOT IMAGES**


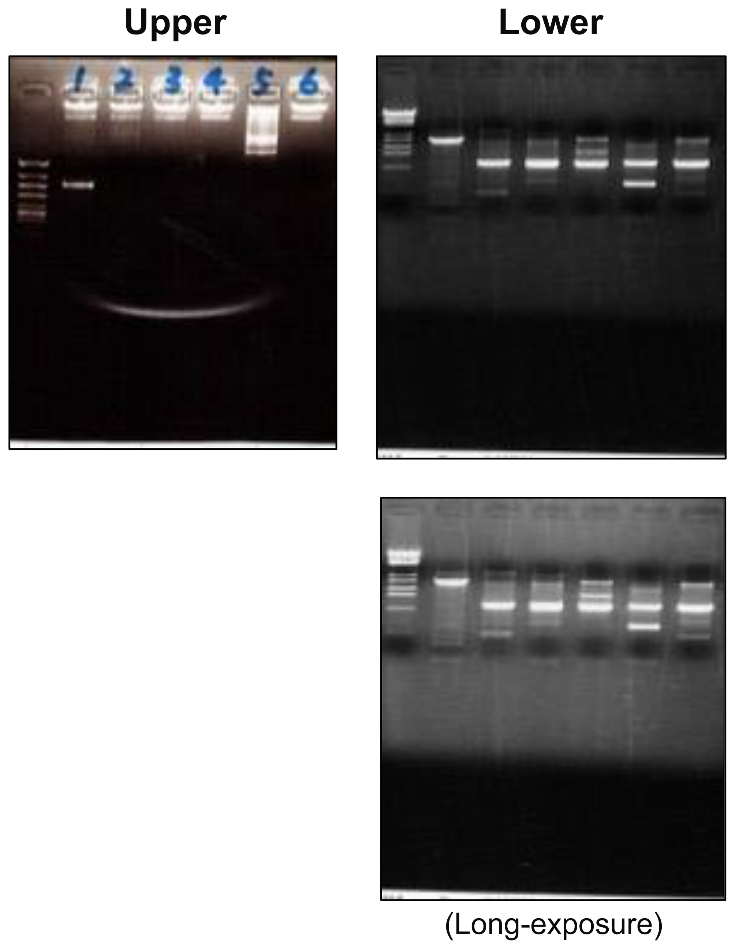


**Supplementary Fig. S4:** This is full gel image of given figure, with figure number Figure 1c. We used λ-StyI (Lower) and φX174-HincII (Upper) as Digested DNA Markers for 0.8% and 2.0% agarose gels, respectively. The λ-StyI yields the following 11 discrete DNA fragments: 19329, 7743, 6223, 4254, 3472, 2690, 1882, 1489, 925, 421, and 74 bp. The φX174-HincII yields the following 13 DNA fragments: 1057, 770, 612, 495, 392, 345, 341, 335, 297, 291, 210, 162, and 79 bp.

**
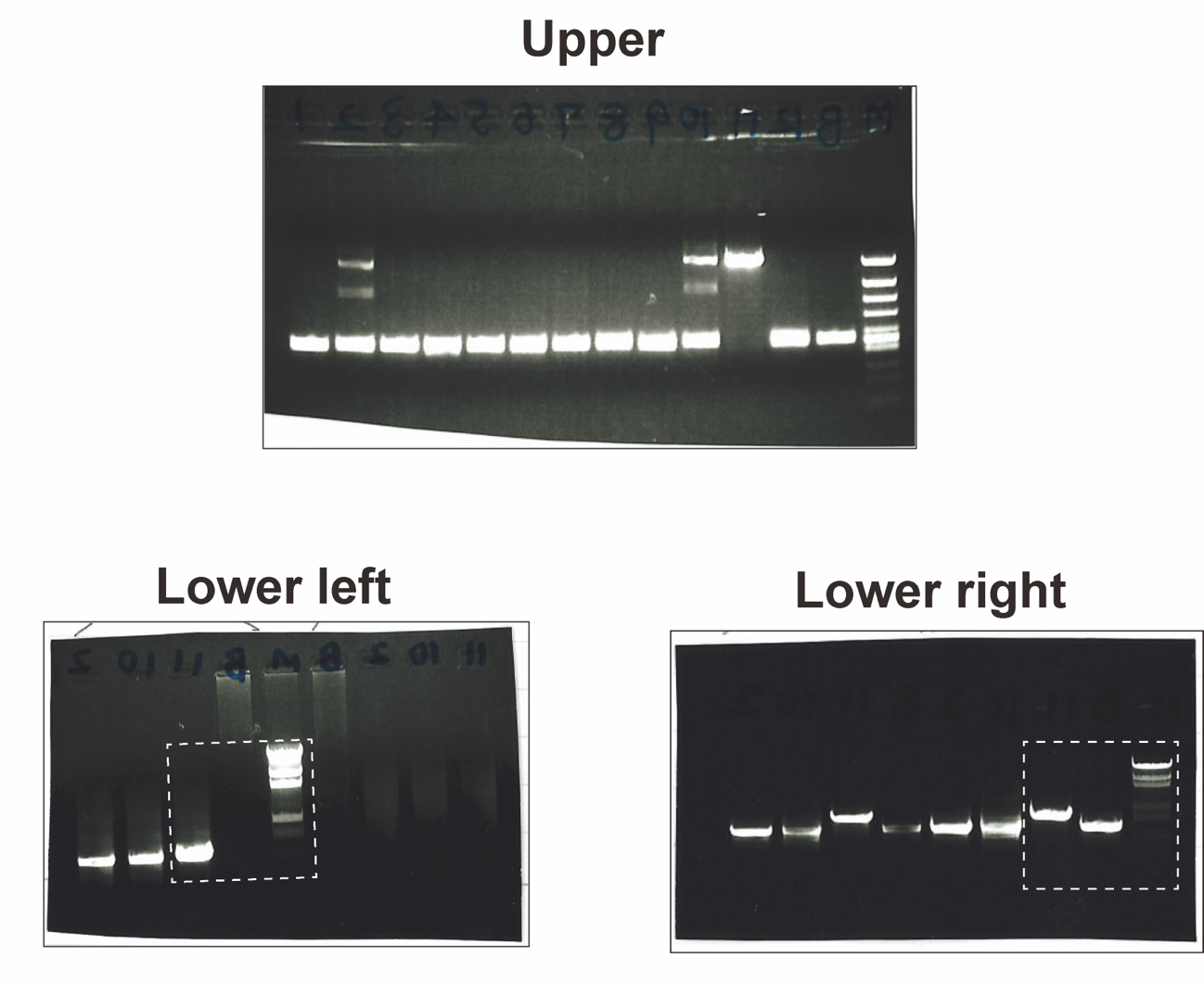
Supplementary Fig. S5:** This is full gel image of given figure, with figure number Figure 2d. Since the photographs were cropped after the image was taken, we do not have a full-length image where the edges of the gel can be visible.


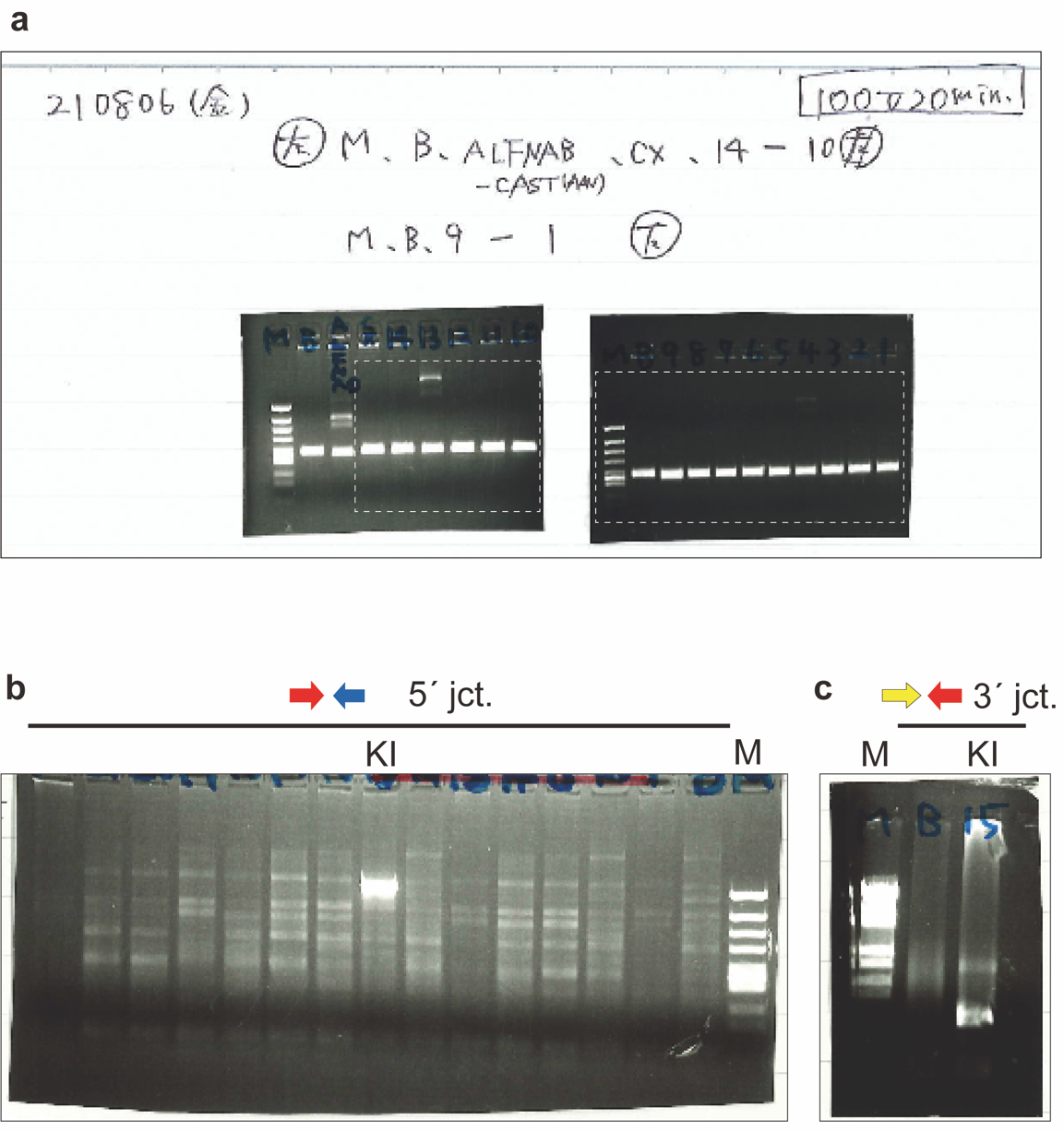


**Supplementary Fig. S6**

**a.** This is full gel image of given figure, with figure number Figure 2e.

**b.** Confirmation of insertions by genomic PCR for *Erc2* locus. 5′ expected band size: 1.1 kb. M, φX174-*Hinc*II digested DNA marker.

**c.** Confirmation of insertions by genomic PCR. 3′ expected band size: 1.5 kb. M, λ-*Sty*I digested DNA marker.


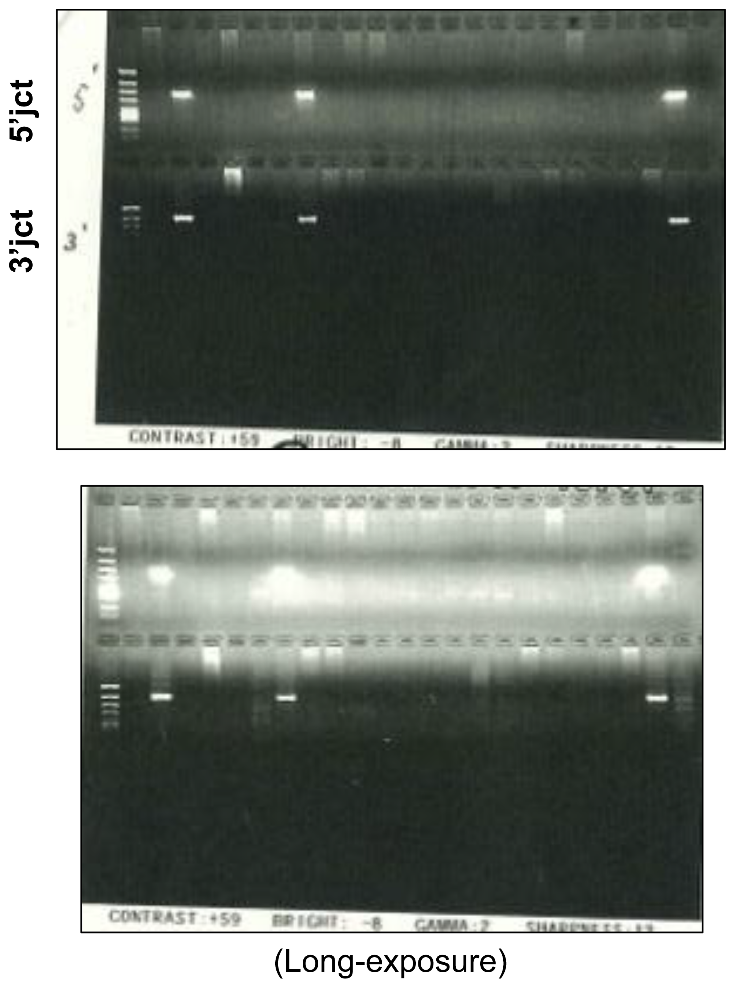


**Supplementary Fig. S7:** This is full gel image of given figure, with figure number Figure 3d.

**
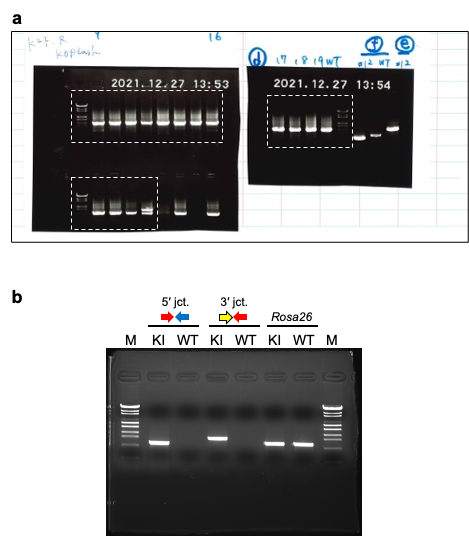
**

**Supplementary Fig. S8**

**a.** This is full gel image of given figure, with figure number Figure 3e.

**b.** Confirmation of insertions by genomic PCR for *Thy1* locus. KI, *Thy1* knock-in rats; WT, wild-type rats. 5′ expected band size: 1.1 kb; 3′ expected band size: 1.5 kb; *Rosa26* expected band size: 1.1 kb. M, λ-*Sty*I digested DNA marker.


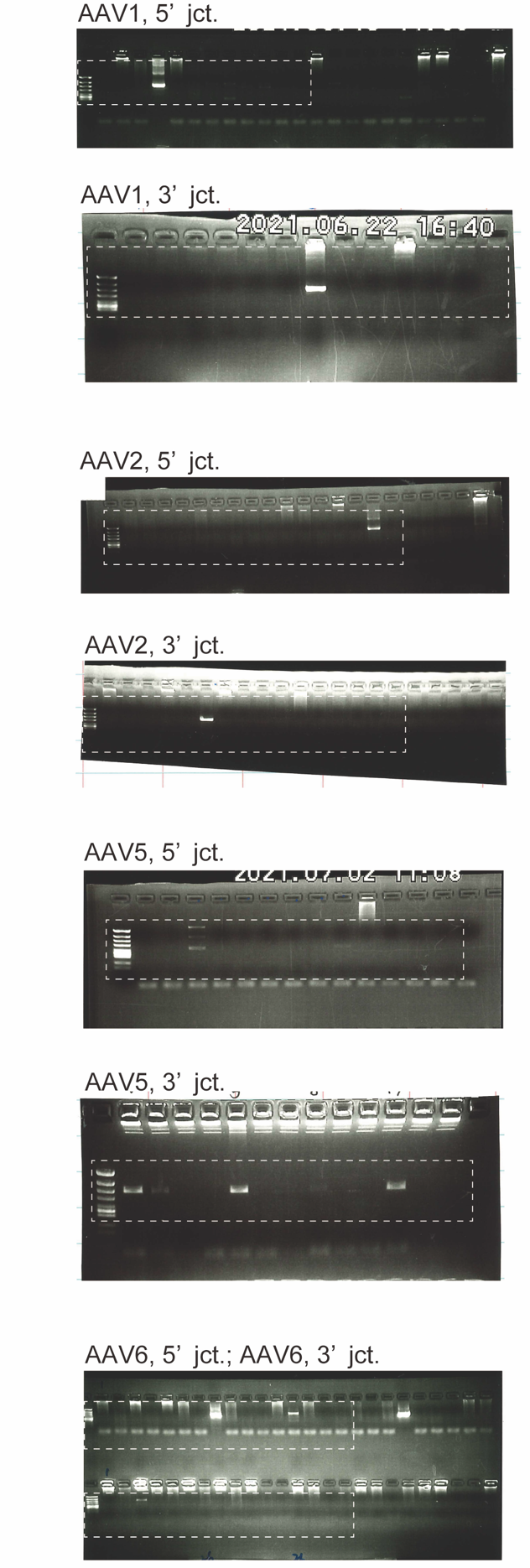
**Supplementary Fig. S9:** This is full gel image of given figure, with figure number Figure S1.


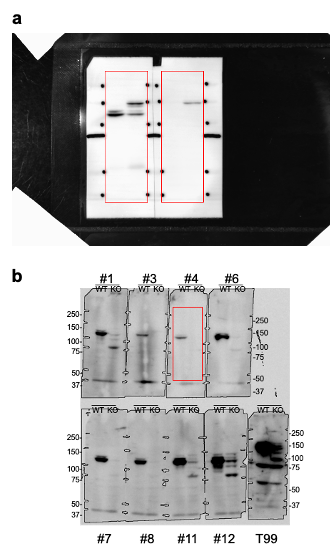
**Supplementary Fig. S10**

**a.** This is full gel image of given figure, with figure number Figure S3b.

**b.** These western blots are screening of monoclonal-ELKS antibodies by using wild type of ELKS cKO mouse brain samples. Clone #4 antibody was used in Figure S3a.
